# Lateral flow nucleic acid biosensor for sensitive detection of microRNAs based on the dual amplification strategy of duplex-specific nuclease and hybridization chain reaction

**DOI:** 10.1371/journal.pone.0185091

**Published:** 2017-09-25

**Authors:** Na Ying, Chuanjing Ju, Xiuwei Sun, Letian Li, Hongbiao Chang, Guangping Song, Zhongyi Li, Jiayu Wan, Enyong Dai

**Affiliations:** 1 Institute of Military Veterinary, Academy of Military Medical Sciences, Changchun, China; 2 East China Sea Fisheries Research Institute, China Academy of Fishery Sciences, Shanghai, China; 3 The General Hospital of FAW, Changchun, China; 4 The Fourth Hospital of Jilin University, Changchun, China; 5 College of Animal Science and Technology, Jilin Agricultural University, Changchun, China; 6 School of Life Science and Technology, Changchun University of Science and Technology, Changchun, China; 7 Heilongjiang Bayi Agricultural University, Daqing, China; 8 Department of Oncology and Hematology, China-Japan Union Hospital of Jilin University, Changchun, China; Gustave Roussy, FRANCE

## Abstract

MicroRNAs (miRNAs) constitute novel biomarkers for various diseases. Accurate and quantitative analysis of miRNA expression is critical for biomedical research and clinical theranostics. In this study, a method was developed for sensitive and specific detection of miRNAs via dual signal amplification based on *duplex specific nuclease* (DSN) and hybridization chain reaction (HCR). A reporter probe (RP), comprising recognition sequence (3’ end modified with biotin) for a target miRNA of miR-21 and capture sequence (5’ end modified with Fam) for HCR product, was designed and synthesized. HCR was initiated by partial sequence of initiator probe (IP), the other part of which can hybridize with capture sequence of RP, and was assembled by hairpin probes modified with biotin (H1-bio and H2-bio). A miR-21 triggered cyclical DSN cleavage of RP, which was immobilized to a streptavidin (SA) coated magnetic bead (MB). The released Fam labeled capture sequence then hybridized with the HCR product to generate a detectable dsDNA. This polymer was then dropped on lateral flow strip and positive result was observed. The proposed method allowed quantitative sequence-specific detection of miR-21 (with a detection limit of 2.1 fM, S/N = 3) in a dynamic range from 100 fM to 100 pM, with an excellent ability to discriminate differences in miRNAs. The method showed acceptable testing recoveries for the determination of miRNAs in serum.

## Introduction

MicroRNAs (miRNAs) are a class of endogenous, short (19−23 nucleotides) single-stranded non-coding RNAs, which serve as critical regulators of gene expression[1]. They play important roles in diverse physiological processes and diseases[2]. Close associations of abnormal miRNA expression and multiple human diseases, especially cancer have been described[3]. MiRNAs are therefore considered potential targets in disease diagnosis and therapy, representing a class of novel biomarkers for diseases such as cancers, and cardiovascular and autoimmune diseases[4]. Thus, simple, rapid, and sensitive miRNA detection methods are urgently needed for further understanding of the biological functions of miRNAs, as well as early disease diagnosis and treatment.

The detection of miRNAs is hampered by their short length, sequence homology among family members, low abundance in total RNA, and susceptibility to degradation[5]. Conventional analytical methods, including Northern blot[6], microarrays and quantitative fluorescence reverse transcription PCR (qRT-PCR)[7], next generation sequencing[8,9] are considered standard methods and widely utilized for miRNA analysis. However, these methods involve elaborate, time-consuming, and expensive processes that require special laboratory equipments[10]. These shortcomings limit their application in point-of-care settings or resource-limited locations. Currently, a variety of novel methods have been developed for miRNA detection, including colorimetric[11,12], fluorescence-based[13,14], bioluminescence-based[15,16], electrochemical[17–19], surface-enhanced Raman spectroscopic[20], surface plasmon resonance[21], Nanopore[22], and mass spectrometric[23] assays. Among them, lateral flow-based colorimetric assay, offers a cost-effective, rapid, and convenient option for miRNA detection with no need of advanced instruments. Nevertheless, lateral flow nucleic acid biosensor (LFNAB) is not satisfactory for miRNA detection due to its poor sensitivity.

The duplex specific nuclease (DSN)-mediated signal amplification strategy was recently developed for miRNAs detection, in which the original detection signal could be amplified linearly without changing target miRNA amounts[10]. DSN displays a high preference for cleaving double-stranded DNA as well as DNA in DNA−RNA hybrid molecule, and is not effective towards single-stranded DNA or single/double-stranded RNA. DSN shows a good ability to discriminate between perfectly and non-perfectly matched duplexes, and does not need special recognition sites[24]. DSN is well suitable for the detection of miRNAs based on the characteristic of DNA cleavage in the DNA−miRNA heteroduplex. Due to its unique enzymatic characteristics and great potential application in miRNA detection[10,25,26], DSN has attracted increasing interest. Despite their remarkable advantages, such as simple methodology, fast experimental protocols, and high specificity, the detection limits of DSN-assisted target recycling methods need further improvement for miRNA detection. This could be achieved by introducing a dual signal amplification process. Lv W et al demonstrated that the DSN-assisted dual signal amplification strategy yields a detection limit as low as 7.3 fM based on DNA/2-OMe-RNA chimeric probes (DR-CPs)[26]. Hao N et al reported a dual target-recycling amplification strategy for sensitive detection of microRNAs based on DSN and catalytic hairpin assembly, with a detection limit as low as 5.4 fM[27]. In spite of the high sensitivity of these two works, the application of electrochemiluminecence and fluorescence measurements requires expensive equipments and materials, which limits their use in low-resource settings or small scale laboratory. And in the latter work, the catalytic hairpin assembly, applied as the second amplification, was initiated by the release of the cleaved probes from magnetic beads, which means that the whole detection time is the sum of two amplification processes. However, comparable to other kit detections, the reagents of which can be prepared in advance, this assay may require more time to complete the whole process.

Hybridization chain reaction (HCR), a signal enhancement technique that depends on the autonomous self-assembly of two metastable hairpin structures (short DNA fragments) through specific interactions, was used to generate a long nicked DNA double helix[28]. Moreover, HCR is an enzyme-free process, and has been widely applied for DNA detection with PCR-like sensitivity[29–31]. Herein, based on miRNA-initiated DNA cleavage by DSN and HCR cascade signal amplification, a lateral flow nucleic acid biosensor for visual and sensitive detection of miRNAs was developed. In the present work, miR-21 was selected as target miRNA. Hybridization chain reaction, served as the second signal amplification, can be prepared in advance or at the same time with the DSN-assisted first amplification, and this saves a tremendous amount of detection time. The proposed strategy could distinguish various homologous sequences containing as little as a single base mismatch. Meanwhile, thanks to the dual signal amplification strategy, the method allows for detection of the target miRNA in a wide dynamic range of 100 fM to 100 pM and has a detection limit as low as 2.1 fM, rendering this method advantageous for analyzing biological samples. Interestingly, this method was successfully applied for analyzing RNA in serum, demonstrating its potential application in clinical sample analysis.

## Materials and methods

### Ethics statement

This study was approved by the Institutional Review Board of Academy of Military Medical Sciences. All the data analyzed in this study were de-identified to protect patient confidentiality.

### Materials and reagents

HPLC-purified microRNAs, oligonucleotide probes were synthesized by Takara Biotechnology Co. Ltd. (Dalian, China). All oligonucleotide sequences are listed in S1 Table. RNase inhibitor and DEPC-treated water were obtained from Sangon Biotech Co., Ltd. (Shanghai, China). Streptavidin-coated magnetic beads (SA-MB, 0.8 mm in diameter, 10 mg/mL) were purchased from Bangs laboratories, Inc. (USA). DSN was obtained from Newborn Co. Ltd. (Shenzhen, China). SA was purchased from Sangon Biotech Co., Ltd. (Shanghai, China). Colloidal AuNPs were from Hualan Chemical Co., Ltd. (Shanghai, China). Anti-Fam antibodies, bovine serum albumin (BSA)-biotin conjugate, were from Ruiqi Biotech Co., Ltd. (Shanghai, China). The serum specimens were obtained from China-Japan Union Hospital of Jilin University, China. All chemicals were from Sigma-Aldrich, Inc. (Saint Louis, MO, USA) and used without further purification.

### Preparation of LFNAB

LFNAB was designed, prepared and assembled as described previously[32], with some modifications. SA was added to 20 nm AuNPs (0.01%, m/v) at a final concentration of 18 μg mL-1 to form colloidal gold particles-streptavidin (AuNPs-SA). The mixture was impregnated on the absorbent pad of the biosensor. The anti-Fam mAb (4 mg mL-1) was coated on the surface of a nitrocellulose (NC) filter membrane at 1 μL/cm as the test line (T line) and BSA-biotin conjugate was coated on the surface of the nitrocellulose membrane at 1 μL/cm as the control line (C line). The concentration of BSA-biotin conjugate was 1.2 mg mL-1. The LFNAB was assembled with the absorbent pad, the NC membrane (in the middle) and absorbent pad and was then cut by the dipstick into 0.5 cm wide, 5 cm long pieces. All the strips were sealed in a plastic bag and stored at room temperature until used.

### Procedure for miRNA detection

The target microRNA triggered and DSN assisted signal amplification was prepared as described previously[26], with some modifications. First, 5μL of the prepared SA-MB was washed thrice, and mixed with 10μL reporter probe (50 nM) at room temperature for 1h. After three times washing steps, 10μL DSN reaction mixture containing 1×DSN buffer (50 mM Tris–HCl pH 8.0, 10 mM MgCl_2_ and 1 mM DTT), 0.2 U DSN, 8 U RNase inhibitor, and different concentrations of target miRNA(the volume of the miRNA sample was 5 μL) was incubated with magnetic beads at 60°C for 60 min.

Next, the MBs were separated away from the reaction mixture using a magnetic separation rack, and the solution (10μL) was transferred to a new centrifuge tube. Then, stop buffer (5μL, 20 mM EDTA) was added into the solution and incubated for 15 min. This was followed by addition of 5μL HCR products containing H1-bio (2μM), H2-bio (2μM) and Initiator Probe (0.4μM) for 2 h. Subsequently, the reaction mixture was incubated at room temperature for 15 min prior to the lateral flow strip assay. Twenty microlitres of the final products were applied to the sample pad. During the assay, the solution migrated upward by capillary force; LFNAB data was read after 10 min. Appearance of visible reddish purple lines in both control and test lines was considered to represent positive target miRNA detection. A negative test result was indicated by a reddish purple line solely at the control line. For quantitative analysis, the signal strength of test/control (T/C) lines (peak areas) of the LFNAB was measured using the Image J software[33].

## Results and discussion

### Principle of the miRNA assay

**Fig 1:**
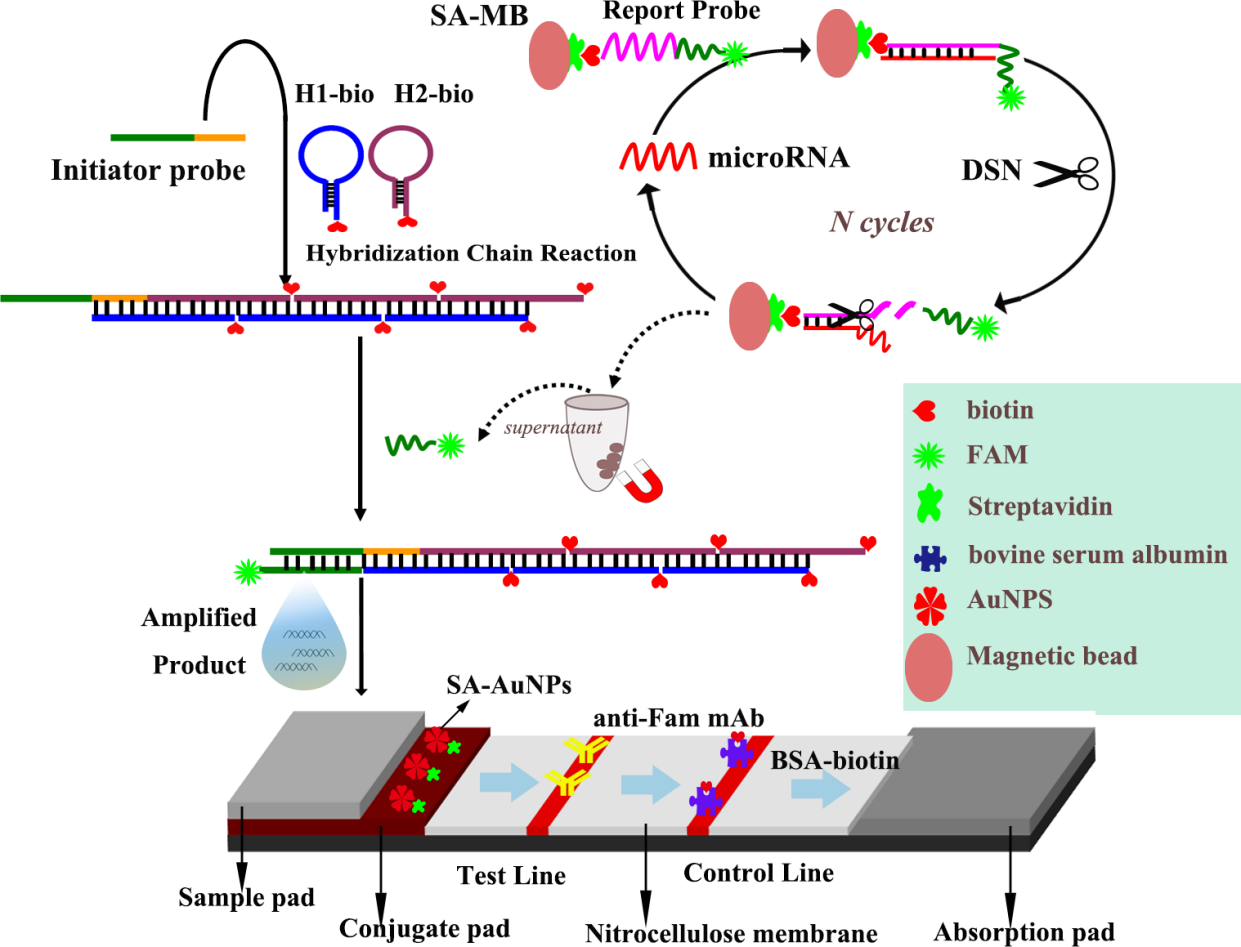
Schematic representation of the dual target-recycling amplification strategy for sensitive detection of microRNAs based on duplex-specific nuclease and hybridization chain reaction with lateral flow assay. The explanations of all acronyms were listed as follows: H1-bio, Hairpin probe1-biotin; SA-MB, Streptavidin-magnetic bead; AuNPs, Au nanoparticles; SA-AuNPs, Streptavidin-Au nanoparticles; BSA, bovine serum albumin; DSN, duplex specific nuclease; FAM, fluorescein isothiocyanates; anti-Fam mAb, anti-FAM- monoclonal antibody.

The principle behind the miRNA assay based on functional magnetic beads and DSN-assisted dual signal amplification is described in detail in Fig 1. A report probe (RP), labeled with a biotin group at the 3′ terminus and a Fam group at the 5′ terminus, consisting of a target miRNA recognition DNA sequence (3′ end) and a capture sequence (5′ end) for HCR products was rationally designed, This probe was first attached to the surface of SA-coated MBs through biotin-SA interaction. Upon addition of target miRNA to the reaction, it hybridized with the report probe to form a RP/miRNA duplex. Once the DNA/RNA heteroduplex was formed, the target recognition sequence was selectively hydrolyzed by DSN. As a result, Fam-labeled capture sequence was released from SA-MB, forming a target-recycling amplification. Afterwards, the MBs together with unreacted RPs were separated away from the reaction mixture using a magnetic separation rack. The supernatant was followed by the addition of biotins labeled HCR polymer and the released capture sequences hybridized with the rest of IP to form Fam-labeled polymers as a signal output.

Visual detection of the formed polymers was performed using the LFNAB. SA-AuNPs and the anti-Fam mAb were pre-immobilized on the conjugate pad and test zone of the LFNAB, respectively. Biotins were pre-immobilized on the control zone. The LFNAB’s sample pad was dipped into the mixture of running buffer and sample solution containing the polymers. When the solution migrated by capillary action and passed the conjugate pad, it rehydrated the SA-AuNP conjugates. The numerous biotin-attached double-helix DNAs of the polymers reacted with SA on the AuNP surface to form HCR-biotin–SA-AuNP complexes, which continued to migrate along the strip. The complexes were then captured on the test zone through specific reactions between the anti-Fam mAb (on test zone) and Fam-labeled initiator sequence of the complexes. AuNP accumulation in the test zone was visualized as a characteristic red band. The excess SA-AuNP conjugates continued to move, and were captured on the control zone via reactions between biotin and SA on the AuNP surface, thus forming a second red band. The presence of target miRNAs was determined by test line’s color, and semi-quantitative detection of the target microRNA was based on the color depth of the detection line. In the absence of target miRNA, RPs were not hydrolyzed by DSN, and no capture sequence fragments were released to capture biotins-labeled HCR polymers; therefore, no red band was observed in the test zone.

### Optimal assay conditions

#### RP concentration

**Fig 2:**
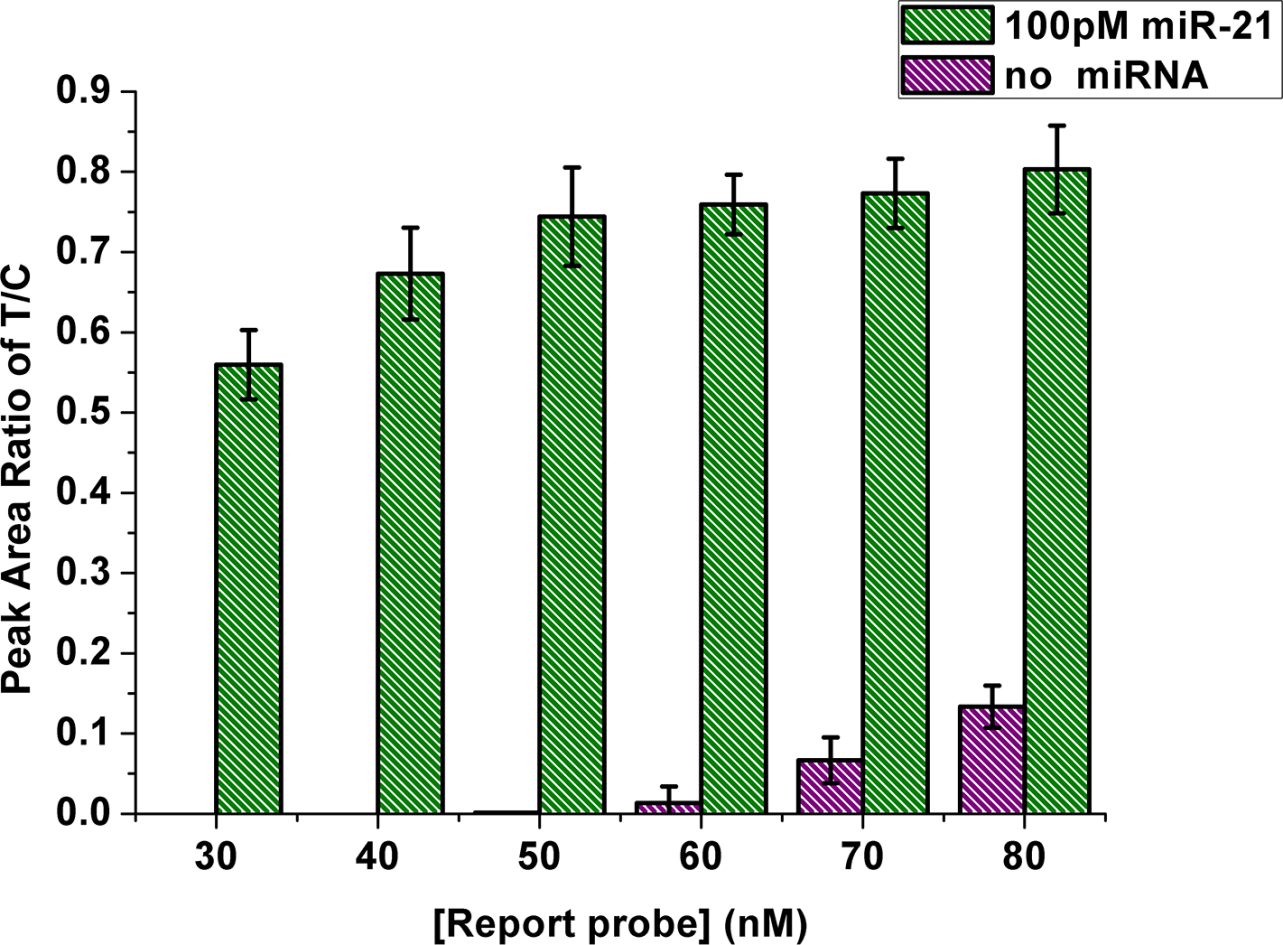
Effect of report probe concentration on response of the strip biosensor. P_T_ and P_C_ are peak areas of test and control lines, respectively. The histograms represent P_T_/P_C_ values in the presence of 100 pM miR-21 (blue) and without miR-21 (purple), respectively. Error bars are standard deviations from three repetitive experiments.

During the experiment, we find that a high concentration of RP results in enhanced hybridization efficiency for HCR, but is also accompanied by a high background signal in the absence of target miRNA. This may be accounted for the instability between the overmuch RP and magnetic beads. Therefore, the concentration of the RP should be optimized. Interestingly, the peak area ratio of test line to control line (P_T_/P_C_) for evaluating miR-21 levels was more precious because it eliminated the effects of immunoreaction dynamics parameters (the efficiencies of antibody-antigen interactions on test line and control line) [34]. As shown in Fig 2, background signals decreased as RP probe concentrations decreased from 80 to 50 nM. At a RP amount of 50 nM, no red line was observed on the strip in the absence of the target. In addition, the signal showed the highest value in the present of the target miRNA (100pM, 5μL). Thus, 50 nM of RP was used in subsequent experiments.

#### DSN temperature

**Fig 3:**
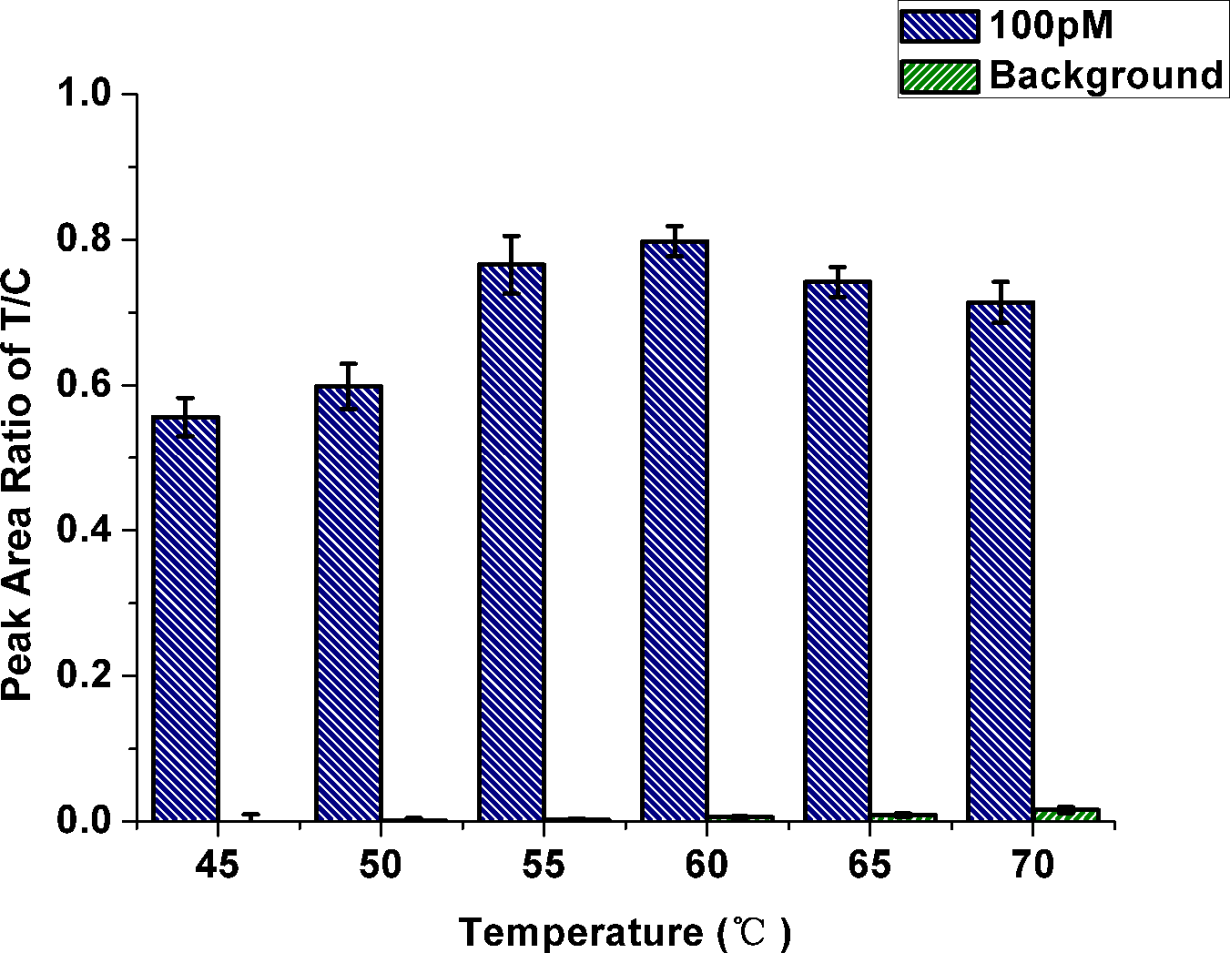
Effect of reaction temperature on response of the strip biosensor. The histograms represent P_T_/P_C_ values in the presence of 100 pM miR-21 (blue) and without miR-21 (green), respectively. P_T_ and P_C_ are peak areas of test and control lines, respectively. Error bars are standard deviations from three independent measurements.

For the whole assay, the reaction temperature is a crucial factor influencing both DSN activity and the stability of RNA/DNA hybrid duplexes. Thus, as an essential experimental factor, the effect of reaction temperature on response of the strip biosensor was first assessed by detecting 100 pM miR-21 at different temperatures (45°C, 50°C, 55°C, 60°C, 65°C and 70°C). A blank sample (without miR-21) at each temperature was setup at the same conditions. As shown in Fig 3, P_T_/P_C_ of the strip increased with reaction temperature ranging between 45°C and 60°C in the presence of 100 pM miR-21, and slightly decreased at 60°C to 70°C (blue histogram in Fig 3). Furthermore, the background (green histogram, no miR-21) was slightly increased with temperature. Therefore, 60°C was considered the optimal reaction temperature.

#### DSN incubation time

**Fig 4:**
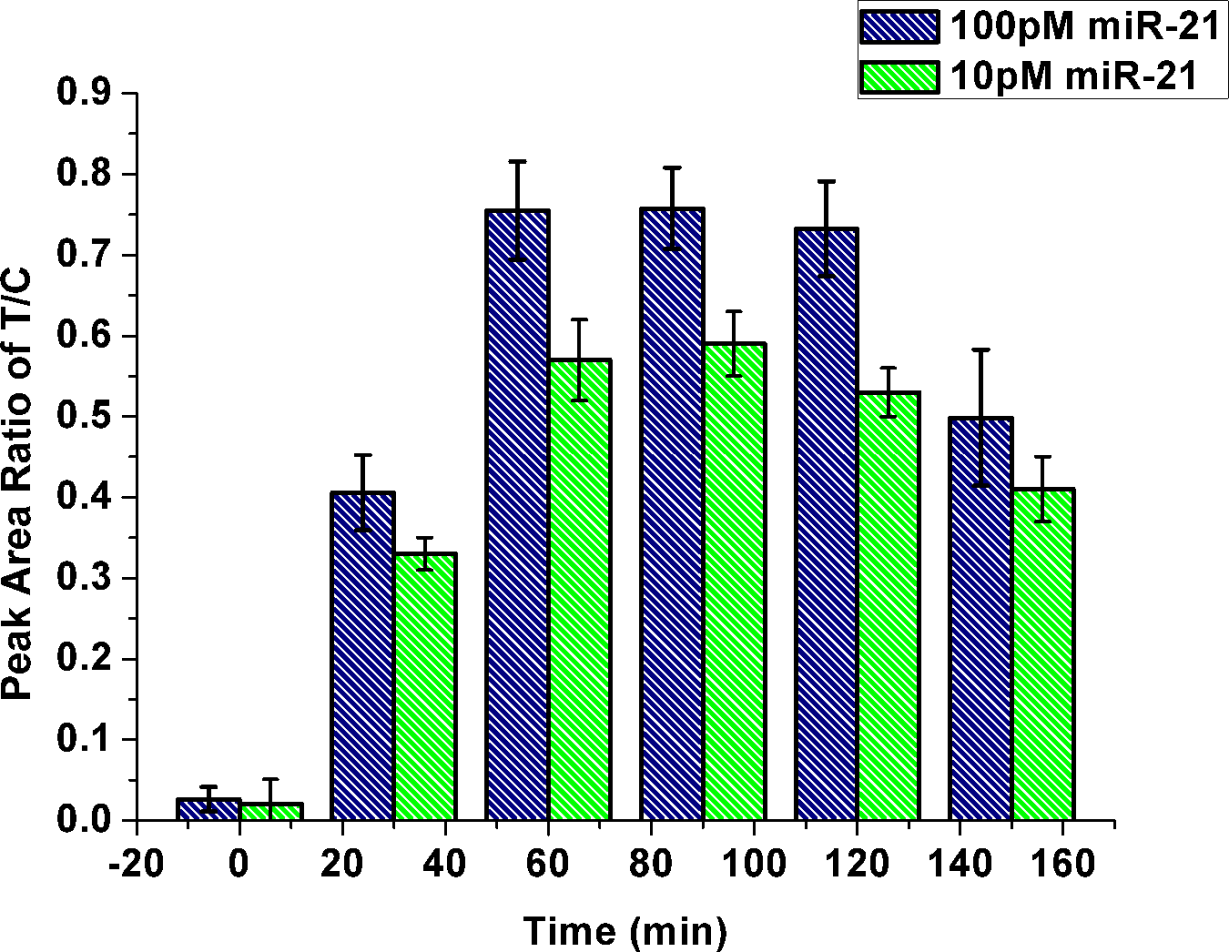
Effect of incubation time on response of the strip biosensor. PT/PC values were obtained by detecting 100 pM miR-21 (blue) and 10 pM miR-21 (green), respectively. Error bars are standard deviations from three independent measurements.

The process of signal amplification was strongly affected by incubation time of DSN. As shown in Fig 4, optical response increased gradually with incubation time (in the presence of 100 pM or 10 pM miR-21) at the early stage, peaking at 60 min; afterward, the same level was maintained, before a decrease occurred at 120 min. When the incubation time was over 120min, the released and FAM-labeled sequence for hybridization with HCR products may be digested, which may lead to the decreased signal. Therefore, 60 min was employed as optimal incubation time.

### Assay sensitivity

**Fig 5:**
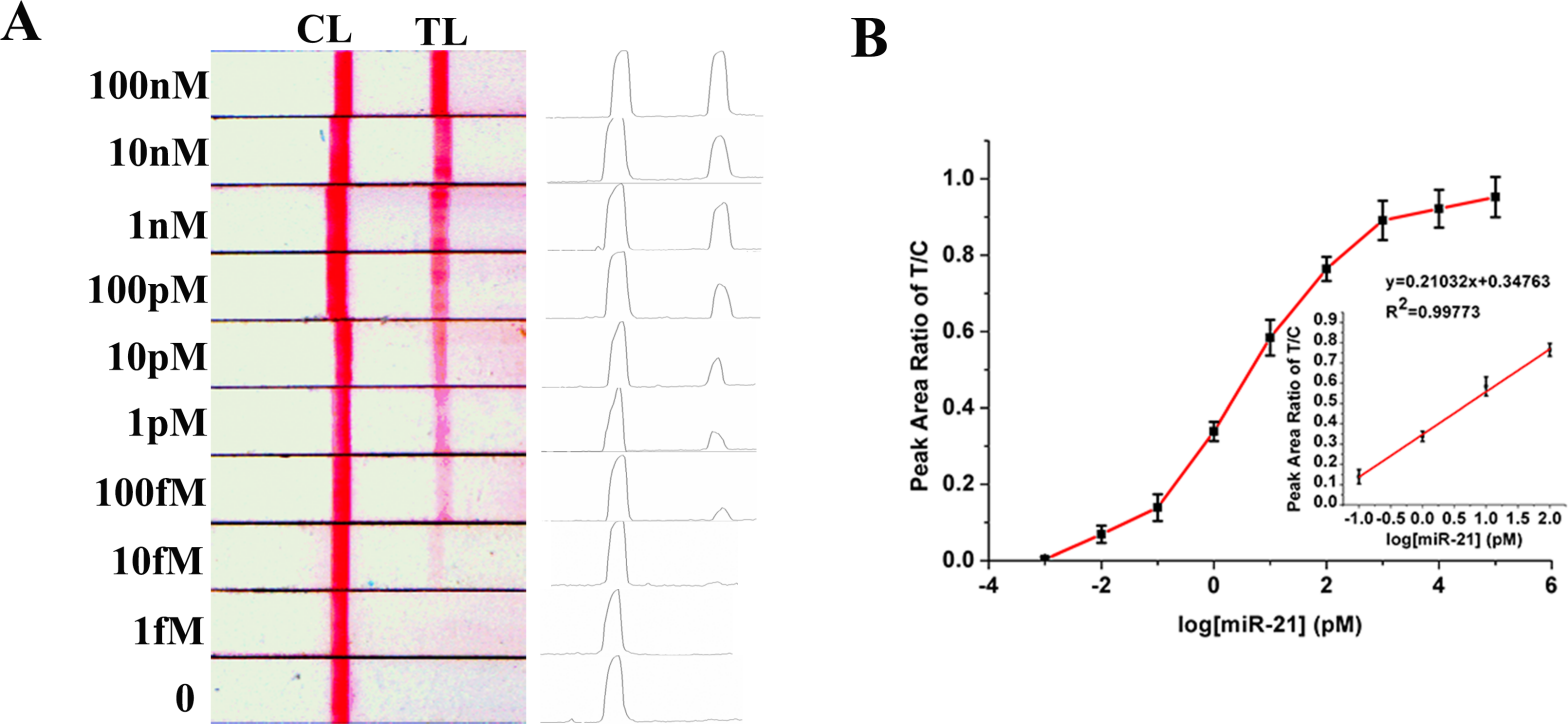
**(A) Photographs of detection results of strips with different miR-21 concentrations. (Left) Images were recorded with a scanner. (Right) Corresponding optical responses of red bands on the strip. Peak areas were analyzed with the Image J software. (B) Calibration curve of miR-21 sensing system.** The curve was plotted as P_T_/P_C_ vs logarithmic value of miR-21 concentration. Inset shows a linear relationship between P_T_/P_C_ and the logarithm of miR-21 concentration. P_T_ and P_C_ are peak areas of test and control lines, respectively. Error bars are standard deviations from three independent measurements.

We subsequently assessed the sensitivity of the proposed method by measuring miR-21 at various concentrations under optimal conditions. As shown in Fig 5(A), test line intensity gradually increased with miR-21 concentrations in the range of 10fM–100 nM(the volume of all target solution was 5μL). Fig 5(B) The logarithmic plot of miR-21 concentration vs. P_T_/P_C_ value was linear in a wide range of miR-21 concentrations, from 100 fM to 100 pM. The calculated limit of detection (LOD) was 2.1 fM according to the rule of three standard deviations. Linear regression analysis of detection data yielded the following equation: P_T_/P_C_ = 0.34763+0.20132 logC, where P_T_/P_C_ is the peak ratio of test line/control line, and C is miRNA-21 concentration in pM.

In this technique, sensitivity was improved by as much as 3 orders of magnitude compared to that of the DNA-gold nanoparticle probe-based assay[10], and 2 orders of magnitude compared to that of the molecular beacon-based method[35]. In addition, the sensitivity of this method was the same order of magnitude as those of dual target-recycling amplification assay based on DSN and catalytic hairpin assembly (LOD:5.4 fM)[27] and DSN-assisted dual signal amplification assay (LOD:7.3 fM)[26]. The high sensitivity achieved by these method mainly relied on cascade amplification and the strong cleavage activity of DSN. However, the above methods use electrochemical and fluorescent assays. These techniques require specific instruments and the test procedures are complicated and are not applicable for POC tests. In the present study, colorimetric analysis is a simple process because samples can be monitored with the naked eye. In addition, colorimetric assays are amenable to point-of-care (POC) testing. The whole procedure can be completed in 150min (HCR products can be prepared in advance).

### Specificity

**Fig 6:**
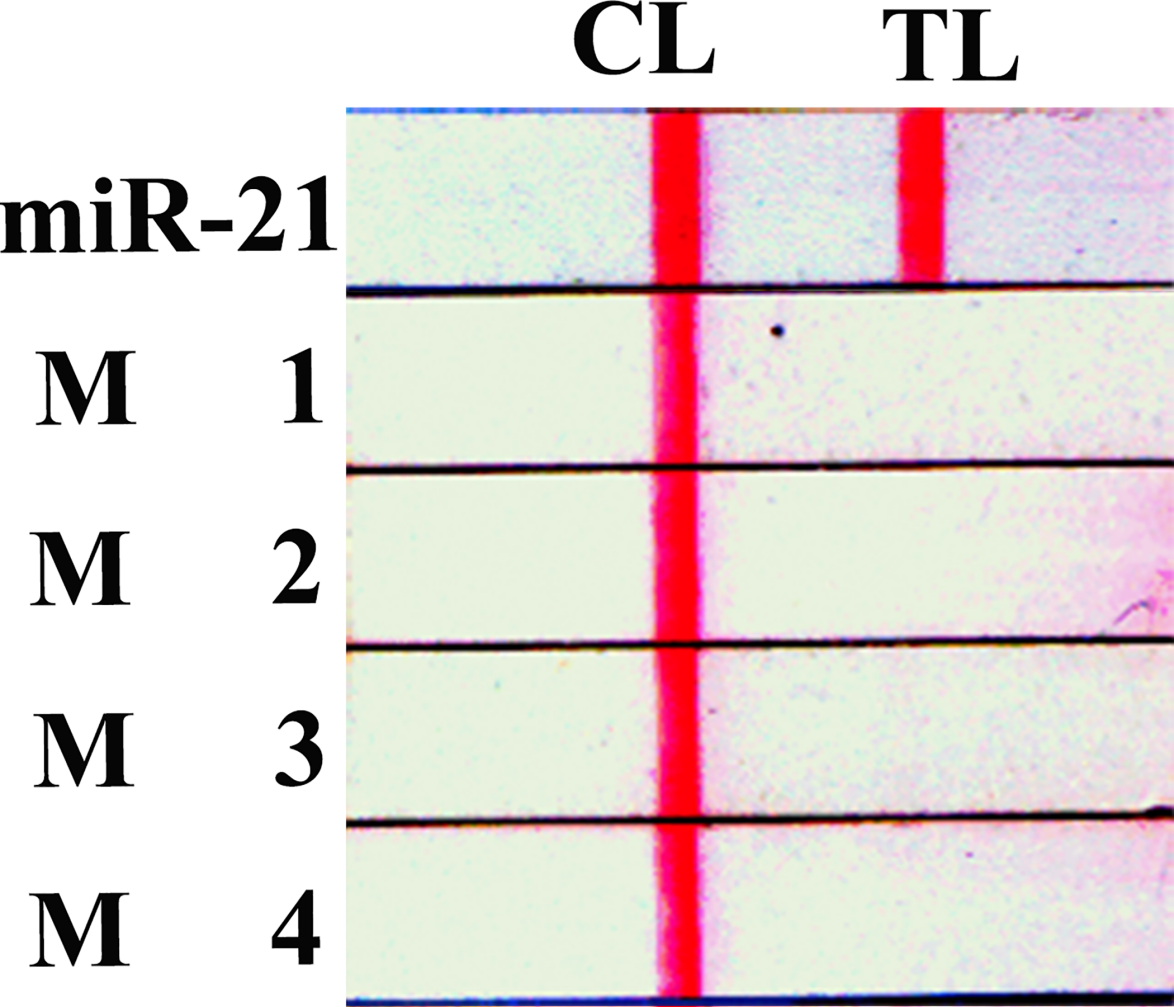
Selectivity of the strip biosensor for miR-21 over competing sequences. Concentrations were 10 nM for miR-21 and 10 nM for other sequences.

Due to short length and sequence similarity among miRNAs, a challenge for miRNA assays is the ability to identify individual members. Specificity of the miRNA assay was evaluated by detecting a target sequence and single-, two, three, and four bases mismatched sequences (S2 Table). After repeated testing, positive results were only obtained with target DNA, while no red line was observed with the mismatch RNAs (Fig 6). These results demonstrated that the proposed technique is particularly attractive for detecting specific miRNA sequences.

### Assays for exogenous miRNA spiked into serum

The commonly-used biosensors are usually inefficient when detecting proteins in complex biological samples (such as serum), since the natural system contain ubiquitous endogenous components producing a high signal background. Here, we assessed the ability of this sensor to detect target miRNA in human serum. Serum was used instead of hybridization buffer to achieve the desired concentrations of the target miR-21 (1, 10, and 100 pM) and other steps were same to the miRNA detection in buffer solution. P_T_/P_C_ was measured in five replicates, and shown in S3 Table as averages and respective RSD percentages. MiR-21 concentration in freshly-collected serum samples was assumed to be zero, because its concentration in serum from non-cancerous individuals is lower than the detection limit obtained in the newly-developed assay. These results showed that the interference of serum could be overcome. This result indicated that this strategy had a promise in practical application with great accuracy and reliability for miRNA detection.

## Conclusion

In summary, lateral flow immunoassay biosensor for detecting miRNAs based on dual amplification strategy of duplex-specific nuclease and hybridization chain reaction offers a versatile platform for rapid, sensitive, and practical detection of miRNAs. First, the whole detection process can be accomplished in 2.5h with simple steps. Second, dual signal amplification greatly increases sensitivity and lower the limit of detection to as low as 2.1 fM. Furthermore, the assay exhibits an excellent discriminatory ability even for highly similar miRNA sequences with a single base difference. Finally, this protocol is successfully implemented for serum samples. Thus, this system is promising for application in biological research and clinical diagnosis.

## Supporting information

S1 Table The sequences used are as follows (5’-3’). In RP, complementary sequence for miR-21 is underlined, capture sequence is italicized; In IP, initiator sequence for HCR is bold, complementary sequence for capture sequence is italicized.
(DOC)

S2 Table The sequences used are as follows (5’-3’). (DOC)

S3 Table Analysis of miR-21 in serum by the strip biosensor. (DOC)

## References

[1] Bartel DP (2004) MicroRNAs: genomics, biogenesis, mechanism, and function. Cell 116: 281–297. 14744438 10.1016/s0092-8674(04)00045-5

[2] Li JB, Tan SB, Kooger R, Zhang CY, Zhang Y (2014) MicroRNAs as novel biological targets for detection and regulation. Chemical Society Reviews 43: 506–517. doi: 10.1039/c3cs60312a 24161958 10.1039/c3cs60312a

[3] Fabian MR, Sonenberg N, Filipowicz W (2010) Regulation of mRNA Translation and Stability by microRNAs. Annual Review of Biochemistry, Vol 79 79: 351–379. doi: 10.1146/annurev-biochem-060308-103103 20533884 10.1146/annurev-biochem-060308-103103

[4] Wang L, Zhu MJ, Ren AM, Wu HF, Han WM, Tan RY, et al (2014) A Ten-MicroRNA Signature Identified from a Genome-Wide MicroRNA Expression Profiling in Human Epithelial Ovarian Cancer. Plos One 9. 10.1371/journal.pone.0096472 PMC4015980 24816756

[5] Labib M, Khan N, Berezovski MV (2015) Protein Electrocatalysis for Direct Sensing of Circulating MicroRNAs. Analytical Chemistry 87: 1395–1403. doi: 10.1021/ac504331c 25495265 10.1021/ac504331c

[6] Houbaviy HB, Murray MF, Sharp PA (2003) Embryonic stem cell-specific MicroRNAs. Developmental Cell 5: 351–358. 12919684 10.1016/s1534-5807(03)00227-2

[7] Shi R, Chiang VL (2005) Facile means for quantifying microRNA expression by real-time PCR. Biotechniques 39: 519–525. 16235564 10.2144/000112010

[8] Chun HYY-JCSWJ-ELHJYSKGPSSLJ (2011) Duplex-specific nuclease efficiently removes rRNA for prokaryotic RNA-seq. Nucleic Acids Research 39. 10.1093/nar/gkr617 PMC3203590 21880599

[9] Miller DFB, Yan PS, Buechlein A, Rodriguez BA, Yilmaz AS, Goel S, et al (2013) A new method for stranded whole transcriptome RNA-seq. Methods 63: 126–134. doi: 10.1016/j.ymeth.2013.03.023 23557989 10.1016/j.ymeth.2013.03.023 PMC3739992

[10] Degliangeli F, Kshirsagar P, Brunetti V, Pompa PP, Fiammengo R (2014) Absolute and Direct MicroRNA Quantification Using DNA-Gold Nanoparticle Probes. Journal of the American Chemical Society 136: 2264–2267. doi: 10.1021/ja412152x 24491135 10.1021/ja412152x

[11] Wu H, Liu YL, Wang HY, Wu J, Zhu FF, Zou P (2016) Label-free and enzyme-free colorimetric detection of microRNA by catalyzed hairpin assembly coupled with hybridization chain reaction. Biosensors & Bioelectronics 81: 303–308. 10.1016/j.bios.2016.03.013 26985582

[12] Li J, Zhang Y, Kuang X, Wang Z, Wei Q (2016) A network signal amplification strategy of ultrasensitive photoelectrochemical immunosensing carcinoembryonic antigen based on CdSe/melamine network as label. Biosens Bioelectron 85: 764–770. doi: 10.1016/j.bios.2016.05.088 27281106 10.1016/j.bios.2016.05.088

[13] He YC, Yin BC, Jiang LH, Ye BC (2014) The rapid detection of microRNA based on p19-enhanced fluorescence polarization. Chemical Communications 50: 6236–6239. doi: 10.1039/c4cc00705k 24788879 10.1039/c4cc00705k

[14] Li RD, Wang Q, Yin BC, Ye BC (2016) Enzyme-free detection of sequence-specific microRNAs based on nanoparticle-assisted signal amplification strategy. Biosensors & Bioelectronics 77: 995–1000. 26547010 10.1016/j.bios.2015.10.082

[15] Cissell KA, Rahimi Y, Shrestha S, Hunt EA, Deo SK (2008) Bioluminescence-based detection of MicroRNA, miR21 in breast cancer cells. Analytical Chemistry 80: 2319–2325. doi: 10.1021/ac702577a 18302417 10.1021/ac702577a

[16] Wang Q, Yin BC, Ye BC (2016) A novel polydopamine-based chemiluminescence resonance energy transfer method for microRNA detection coupling duplex-specific nuclease-aided target recycling strategy. Biosensors & Bioelectronics 80: 366–372. 26866561 10.1016/j.bios.2016.02.005

[17] Campuzano S, Pedrero M, Pingarron JM (2014) Electrochemical genosensors for the detection of cancer-related miRNAs. Analytical and Bioanalytical Chemistry 406: 27–33. doi: 10.1007/s00216-013-7459-z 24247551 10.1007/s00216-013-7459-z

[18] Chen AY, Ma SY, Zhuo Y, Chai YQ, Yuan R (2016) In Situ Electrochemical Generation of Electrochemiluminescent Silver Naonoclusters on Target-Cycling Synchronized Rolling Circle Amplification Platform for MicroRNA Detection. Analytical Chemistry 88: 3203–3210. doi: 10.1021/acs.analchem.5b04578 26885698 10.1021/acs.analchem.5b04578

[19] Jamali AA, Pourhassan-Moghaddam M, Dolatabadi JEN, Omidi Y (2014) Nanomaterials on the road to microRNA detection with optical and electrochemical nanobiosensors. Trac-Trends in Analytical Chemistry 55: 24–42.

[20] Driskell JD, Seto AG, Jones LP, Jokela S, Dluhy RA, Zhao YP, et al (2008) Rapid microRNA (miRNA) detection and classification via surface-enhanced Raman spectroscopy (SERS). Biosensors & Bioelectronics 24: 917–922. 10.1016/j.bios.2008.07.060 18799303

[21] Ding XJ, Yan YR, Li SQ, Zhang Y, Cheng W, Cheng Q, et al (2015) Surface plasmon resonance biosensor for highly sensitive detection of microRNA based on DNA super-sandwich assemblies and streptavidin signal amplification. Analytica Chimica Acta 874: 59–65. doi: 10.1016/j.aca.2015.03.021 25910447 10.1016/j.aca.2015.03.021

[22] Zhang XY, Wang Y, Fricke BL, Gu LQ (2014) Programming Nanopore Ion Flow for Encoded Multiplex MicroRNA Detection. Acs Nano 8: 3444–3450. doi: 10.1021/nn406339n 24654890 10.1021/nn406339n PMC4004327

[23] Xu FF, Yang T, Chen Y (2016) Quantification of microRNA by DNA-Peptide Probe and Liquid Chromatography-Tandem Mass Spectrometry-Based Quasi-Targeted Proteomics. Analytical Chemistry 88: 754–763. doi: 10.1021/acs.analchem.5b03056 26641144 10.1021/acs.analchem.5b03056

[24] Shagin DA, Rebrikov DV, Kozhemyako VB, Altshuler IM, Shcheglov AS, Zhulidov PA, et al (2002) A novel method for SNP detection using a new duplex-specific nuclease from crab hepatopancreas. Genome Research 12: 1935–1942. doi: 10.1101/gr.547002 12466298 10.1101/gr.547002 PMC187582

[25] Yin BC, Liu YQ, Ye BC (2012) One-Step, Multiplexed Fluorescence Detection of microRNAs Based on Duplex-Specific Nuclease Signal Amplification. Journal of the American Chemical Society 134: 5064–5067. doi: 10.1021/ja300721s 22394262 10.1021/ja300721s

[26] Lv W, Zhao J, Situ B, Li B, Ma W, Liu J, et al (2016) A target-triggered dual amplification strategy for sensitive detection of microRNA. Biosens Bioelectron 83: 250–255. doi: 10.1016/j.bios.2016.04.053 27131998 10.1016/j.bios.2016.04.053

[27] Hao N, Dai PP, Yu T, Xu JJ, Chen HY (2015) A dual target-recycling amplification strategy for sensitive detection of microRNAs based on duplex-specific nuclease and catalytic hairpin assembly. Chem Commun (Camb) 51: 13504–13507. 26216522 10.1039/c5cc05350a

[28] Pierce RMDaNA (2004) Triggered amplification by hybridization chain reaction PNAS 101: 4. 15492210 10.1073/pnas.0407024101 PMC524468

[29] Liu P, Yang XH, Sun S, Wang Q, Wang KM, Huang J, et al (2013) Enzyme-Free Colorimetric Detection of DNA by Using Gold Nanoparticles and Hybridization Chain Reaction Amplification. Analytical Chemistry 85: 7689–7695. doi: 10.1021/ac4001157 23895103 10.1021/ac4001157

[30] Ma CP, Wang WS, Mulchandani A, Shi C (2014) A simple colorimetric DNA detection by target-induced hybridization chain reaction for isothermal signal amplification. Analytical Biochemistry 457: 19–23. doi: 10.1016/j.ab.2014.04.022 24780220 10.1016/j.ab.2014.04.022

[31] Li CX, Wang HY, Shen J, Tang B (2015) Cyclometalated Iridium Complex-Based Label-Free Photoelectrochemical Biosensor for DNA Detection by Hybridization Chain Reaction Amplification. Analytical Chemistry 87: 4283–4291. doi: 10.1021/ac5047032 25816127 10.1021/ac5047032

[32] Yin R, Sun YJ, Yu S, Wang Y, Zhang MP, Xu YW, et al (2016) A validated strip-based lateral flow assay for the confirmation of sheep-specific PCR products for the authentication of meat. Food Control 60: 146–150.

[33] Schneider CA, Rasband WS, Eliceiri KW (2012) NIH Image to ImageJ: 25 years of image analysis. Nature Methods 9: 671–675. 22930834 10.1038/nmeth.2089 PMC5554542

[34] Deng SL, Shan S, Xu CL, Liu DF, Xiong YH, Wei H, et al (2014) Sample Preincubation Strategy for Sensitive and Quantitative Detection of Clenbuterol in Swine Urine Using a Fluorescent Microsphere-Based Immunochromatographic Assay. Journal of Food Protection 77: 1998–2003. doi: 10.4315/0362-028X.JFP-14-086 25364937 10.4315/0362-028X.JFP-14-086

[35] Lin XY, Zhang C, Huang YS, Zhu Z, Chen X, Yang CJ (2013) Backbone-modified molecular beacons for highly sensitive and selective detection of microRNAs based on duplex specific nuclease signal amplification. Chemical Communications 49: 7243–7245. doi: 10.1039/c3cc43224f 23842896 10.1039/c3cc43224f

