# Supplementary material for: Lateral flow nucleic acid biosensor for sensitive detection of microRNAs based on the dual amplification strategy of duplex-specific nuclease and hybridization chain reaction
Source: PLoS One. 2017 Sep 25;12(9):e0185091. doi: 10.1371/journal.pone.0185091 (PMC5612651; doi:10.1371/journal.pone.0185091)
Supplement: S1 Table — In RP, complementary sequence for miR-21 is underlined, capture sequence is italicized; In IP, initiator sequence for HCR is bold, complementary sequence for capture sequence is italicized. (DOC) [file pone.0185091.s001.doc]

**S1 Table.**

| Name | Abbreviation | ­Sequence (5’－3’) |
| --- | --- | --- |
| miRNA-21 | miR-21 | UAGCUUAUCAGACUGAUGUUGA |
| Reporter probe | RP | Fam-TTTTTT*TTAATGCTAATC*TCAACATCAGTCTGATAAGCTATTT-Biotin |
| Initiator probe | IP | *GATTAGCATTAA***AGTCTAGGATTCGGCGTGGGTTAA** |
| Hairpin1-Biotin | H1-Biotin | Biotin-TTTTTAACCCACGCCGAATCCTAGACT**CAAAGT**AGTCTAGGATTCGGCGTG |
| Hairpin2-Biotin | H2- Biotin | AGTCTAGGATTCGGCGTGGGTTAACACGCCGAATCCTAGACTACTTTGTTT-Biotin |
