# Supplementary material for: Lateral flow nucleic acid biosensor for sensitive detection of microRNAs based on the dual amplification strategy of duplex-specific nuclease and hybridization chain reaction
Source: PLoS One. 2017 Sep 25;12(9):e0185091. doi: 10.1371/journal.pone.0185091 (PMC5612651; doi:10.1371/journal.pone.0185091)
Supplement: S2 Table — (DOC) [file pone.0185091.s002.doc]

**S2 Table.**

| Name | Abbreviation | ­Sequence (5’－3’) |
| --- | --- | --- |
| miRNA-21 | miRNA-21 | UAGCUUAUCAGACUGAUGUUGA |
| One base mismatched | M1 | UAGCUUAUCACACUGAUGUUGA |
| Two base mismatched | M2 | UAGCUUAUCACACUGAAGUUGA |
| Three base mismatched | M3 | UAGCUUAACAGACUGAACUUGA |
| Four base mismatched | M4 | UAGCUUAACACACUGAACUUGA |
| miRNA-141 | miRNA-141 | CAUCUUCCAGUACAGUGUUGGA |
