# Supplementary material for: Lateral flow nucleic acid biosensor for sensitive detection of microRNAs based on the dual amplification strategy of duplex-specific nuclease and hybridization chain reaction
Source: PLoS One. 2017 Sep 25;12(9):e0185091. doi: 10.1371/journal.pone.0185091 (PMC5612651; doi:10.1371/journal.pone.0185091)
Supplement: S3 Table — (DOC) [file pone.0185091.s003.doc]

**S3 Tabl**e.

| Sample number | Added miR-21/pM | Detected miR-21/pM | Recovery (%) (n=5) | RSD (%) |
| --- | --- | --- | --- | --- |
| 1 | 100 | 96.4 | 96.4 | 5 |
| 2 | 10 | 9.73 | 97.3 | 3 |
| 3 | 1 | 0.941 | 94.1 | 3 |
